# Chronically hypertensive transgenic mice expressing human AT1R haplotype-I exhibit increased susceptibility to *Francisella tularensis*

**DOI:** 10.3389/fmicb.2023.1173577

**Published:** 2023-05-17

**Authors:** Harshada Ketkar, Maha Alqahtani, Samantha Tang, Sreema Puthiya Parambath, Chandra Shekhar Bakshi, Sudhir Jain

**Affiliations:** Department of Pathology, Microbiology and Immunology, New York Medical College, Valhalla, NY, United States

**Keywords:** *Francisella*, aging, transgenic, hypertension, inflammation, sepsis, cytokine storm, respiratory tularemia

## Abstract

Age-related illnesses, including hypertension and accompanying metabolic disorders, compromise immunity and exacerbate infection-associated fatalities. Renin-angiotensin system (RAS) is the key mechanism that controls blood pressure. Upregulation of RAS through angiotensin receptor type 1 (AT1R), a G-protein coupled receptor, contributes to the pathophysiological consequences leading to vascular remodeling, hypertension, and end-organ damage. Genetic variations that increase the expression of human AT1R may cause the above pathological outcomes associated with hypertension. Previously we have shown that our chronically hypertensive transgenic (TG) mice containing the haplotype-I variant (Hap-I, hypertensive genotype) of human *AT1R* (*hAT1R*) gene are more prone to develop the metabolic syndrome-related disorders as compared to the TG mice containing the haplotype-II variant (Hap-II, normotensive genotype). Since aging and an increased risk of hypertension can impact multiple organ systems in a complex manner, including susceptibility to various infections, the current study investigated the susceptibility and potential effect of acute bacterial infection using a Gram-negative intracellular bacterial pathogen, *Francisella tularensis* in our hAT1R TG mice. Our results show that compared to Hap-II, *F. tularensis-*infected aged Hap-I TG mice have significantly higher mortality post-infection, higher bacterial load and lung pathology, elevated inflammatory cytokines and altered gene expression profile favoring hypertension and inflammation. Consistent with worsened phenotype in aged Hap-I mice post-*Francisella* infection, gene expression profiles from their lungs revealed significantly altered expression of more than 1,400 genes. Furthermore, bioinformatics analysis identified genes associated with RAS and IFN-γ pathways regulating blood pressure and inflammation. These studies demonstrate that haplotype-dependent over-expression of the *hAT1R* gene leads to enhanced susceptibility and lethality due to *F. tularensis* LVS infection, which gets aggravated in aged animals. Clinically, these findings will help in exploring the role of AT1R-induced hypertension and enhanced susceptibility to infection-related respiratory diseases.

## Introduction

The severity of an infectious disease is primarily associated with an effect of pathogen or host factors. Underlying comorbidities like diabetes, hypertension, obesity, cardiovascular, renal, and chronic lung diseases have the worst outcomes. These comorbidities, mostly associated with old age, compromise immunity, exacerbate organ damage, and may result in death. The immune mechanisms contributing to enhanced susceptibility to infections in an aged individual with these underlying conditions are not very well known. Hypertension is one of the major predisposing factors responsible for increased susceptibility to infections (Harrison et al., 2011). The renin-angiotensin-aldosterone system (RAAS) plays a major role in blood pressure regulation (Muñoz-Durango et al., 2016; Mirabito Colafella et al., 2019). Angiotensin receptor type 1 (AT1R), a G-protein coupled receptor, mediates the effect of angiotensin-II and contributes to pathophysiological consequences such as vascular remodeling, hypertension, and end-organ damage (Mehta and Griendling, 2007; Forrester et al., 2018). Genetic variations that increase human *AT1R* (*hAT1R*) expression can lead to these pathological outcomes due to RAAS overactivity. In previous studies, we have developed unique transgenic (TG) mouse models that express the human *AT1R* (*hAT1R*) gene (Jain et al., 2013). Mice TG for haplotype-I (Hap-I) of *hAT1R* are hypertensive, while those with haplotype-II (Hap-II) are normotensive and exhibit normal blood pressure. Using our Hap-I TG mouse model, we have demonstrated that aging is associated with an enhanced trans-regulation of the *hAT1R* gene *via* stronger binding of STAT3, USF1, and Pol-II transcription factors (Jain et al., 2018b). We have also shown that higher *hAT1R* expression leads to elevated blood pressure and age-associated metabolic complications in Hap-I TG mice (Jain et al., 2018a).

*Francisella tularensis* is a Gram-negative, intracellular bacterial pathogen responsible for causing an acutely fatal disease known as tularemia (Ellis et al., 2002; Sjöstedt, 2007). *F. tularensis* is highly virulent and only 1–10 organisms, if inhaled, are sufficient to cause severe disease and death. Due to its extremely high virulence, *Francisella* was used in bioweapon programs in the past and now poses a threat as a potential bioterror agent (Altman and Wachs, 2002). The Centers for Disease Control has categorized *F. tularensis* as a Tier 1 Category A select agent. Of the four subspecies of *F. tularensis,* subspecies *tularensis* and *holarctica* are capable of causing tularemia in humans. The live vaccine strain (LVS) derived from the *F. tularensis* subspecies *holarctica* serves as an excellent surrogate for the highly virulent *F. tularensis* subspecies *tularensis* strains to study the immunopathogenesis of tularemia in a mouse model (Mörner et al., 1993). The clinical manifestations of tularemia depend on the route of bacterial entry and may present in ulceroglandular, typhoidal, or pneumonic form if the bacteria are acquired through the skin, orally, or through inhalation. Pneumonic tularemia is an acute and fatal form of the disease (Sjöstedt, 2007). An increased incidence of tularemia has been reported in several mid-western states of the United States in the past decade. A higher incidence of tularemia is reported among white males of all age groups. However, individuals over the age of 65 mainly exhibit the highest incidence of tularemia in the United States (Bishop et al., 2023).

Although hypertension is considered a major risk factor for diminished immunity and increased susceptibility to infections, the exact pathophysiology of an infectious disease in an aged hypertensive model is not well studied. In this study, we used pneumonic tularemia as an infectious disease model and Hap-I and Hap-II TG mice to study the impact of hypertension and old age on susceptibility and immunopathogenesis of *F. tularensis* infection. Our results identify unique immunopathogenic mechanisms that may contribute to the increased susceptibility of aged hypertensive individuals to acute infectious diseases.

Initially, we looked at the dose-dependent effect of intranasal (i.n.) *F. tularensis* LVS infection on morbidity and mortality in our transgenic strains. We also studied the lung pathology and pro-inflammatory cytokine profile in the serum and tissue post-*F. tularensis* LVS infection. RNA sequencing was performed to analyze alterations in gene expression profiles in canonical pathways related to hypertension and inflammation. Our results demonstrate increased susceptibility of hypertensive, aged Hap-I TG mice to *F. tularensis* LVS infection as evidenced by increased mortality; enhanced lung pathology and bacterial burden; higher levels of cytokines, and an upregulated expression of the components of the RAS pathway.

## Materials and methods

### Bacterial strains and media

The *F. tularensis* subsp. *holarctica* LVS (ATCC 29684; American Type Culture Collection, Rockville, MD) was used in this work. All the work was carried out under biosafety level 2 (BSL2) containment conditions. All bacterial stock cultures were grown at 37°C with 5% CO_2_ on Mueller-Hinton (MH) chocolate agar plates. After 48 h of growth, individual colonies were inoculated into MH broth (MHB) supplemented with anhydrous calcium chloride, hydrous magnesium chloride, glucose, ferric pyrophosphate, and isovitalex (BD Biosciences, San Jose, CA). The cultures were grown at 37°C for 12–16 h with constant shaking. Aliquots of mid-log-phase bacteria grown in MHB were stored at −80°C. Before use, the frozen vial of bacterial stock culture was thawed in a water bath, subculture and grown at 37°C with 5% CO_2_ on Mueller-Hinton (MH) chocolate agar plates for 12–16 h.

### *In vivo* studies

Transgenic mice with either Hap-I (hypertensive) or Hap-II (normotensive) haplotypes were generated as described previously (Jain et al., 2013). Briefly, the *hAT1R* BAC plasmid was modified by replacing the 1.7-kb promoter region from the DNA of a subject containing hap-I of the hAT1R gene. Hap-II was generated from Hap-I by replacing a 1.2-kb promoter fragment from a human subject containing Hap-II of the *hAT1R* gene. These BAC plasmids were linearized by *Not*I and microinjected in mice. The blood pressure measurement in the conscious state by radio telemetry confirmed the hypertensive phenotype of Hap-I and normal blood pressure of Hap-II transgenic lines. Adult (12–16 weeks) and old (72–80 weeks) *AT1R* Hap-I and Hap-II mice were maintained in the animal facility of New York Medical College. Mice were administered an anesthetic cocktail consisting of ketamine (5 mg/kg) and xylazine (4 mg/kg) intraperitoneally and underwent experimental manipulation only after they failed to exhibit a toe pinch reflex. The deeply anesthetized mice were infected intranasally (i.n.) with 1 × 10^4^ CFU or 1 × 10^5^ CFUs of *F. tularensis* LVS. The bacterial infection dose was quantified by plating serial dilutions of inoculum onto MH chocolate agar plates. Mice were observed for a period of 15 days for morbidity and mortality. The body weights of all mice were recorded daily post-infection to monitor morbidity. Lung tissues on days three and five post-infection were homogenized in 1 mL of PBS, centrifuged, and the supernatant was used to quantify the bacterial burden as Log_10_ CFU/mL. All animal procedures were conducted in accordance with the Institutional Animal Care and Use Committee (IACUC) guidelines approved by New York Medical College.

### Histology

Lung and spleen of control and *F. tularensis* LVS-infected mice were fixed in 10% neutral buffered formalin, paraffin embedded, and 3-micron sections were prepared using Leica Microtome. The sections were stained with hematoxylin–eosin (H & E) and the images were captured at 20× magnification using a Nikon Eclipse 90i microscope (Nikon, United States).

### Cytokine analysis

The blood from *F. tularensis*-infected mice was collected on day three and day five post-infection by retro-orbital bleeding. The serum was separated and used for cytokine analysis as per the manufacturer’s instructions (Quantibody^®^ Mouse Cytokine Array-Ray Biotech, GA).

### RNA extraction and qRT-PCR analysis

The left lobe of the lung collected from control and *F. tularensis-*infected mice on days zero and five post-infection was stored in RNA Later at −80°C. RNA was isolated using an RNAeasy Kit (Qiagen) from each lung sample. Two hundred nanograms of the total RNA was converted to cDNA using the High-Capacity cDNA Reverse Transcription Kit (Applied biosystem, Thermo Fisher Scientific). The quantitative real-time PCRs (qRT-PCR) were performed using the QuantStudio 3 Real-Time PCR System (ThermoFisher), with 40 cycles of denaturation (95°C for 15 s), annealing (55°C for 30s), and extension (72°C for 30s). Primers of human *AT1R* and mouse *At1ar, Stat3, Usf, Ace, Ccl2, Tnf-α, Il-17, Ifn-γ,* and *Il-6* were purchased from Integrated DNA Technologies (IDT, IA).

### RNA-sequencing and gene expression analysis

RNA isolated from the lungs of control and *F. tularensis* LVS-infected mice on day five post-infection was quality was checked by 4200 TapeStation (Agilent Technologies, Santa Clara, CA) and RNA quantity was determined by Qubit 2 fluorometer (Life Technologies, Carlsbad, CA). For each sample, 400 ng of RNA was used to construct a cDNA sequencing library using the TruSeq Stranded Total RNA Library Preparation Kit (Illumina, San Diego, CA) in accordance with the manufacturer’s protocol using the polyadenylated RNA extraction. Sequencing of paired end reads (75 bp × 2) was performed in Illumina NextSeq 550 system.

Raw sequence reads were de-multiplexed and trimmed for adapters by using the Illumina bcl2fastq conversion software (v2.19). Sequence reads of each sample were pseudo-aligned to Ensembl mouse GRCm38 (release 100) reference transcriptome and the gene transcript abundance was quantified by using Kallisto (v0.43.1) algorithm (Bray et al., 2016). The differential expression of genes and transcripts was achieved in paired groups by using DESeq2 package (doi:10.1186/s13059-014-0550-8) in the RStudio platform (v1.3.1073, with R v4.1.0). The gene-enriched gene ontology and pathway analyses were achieved by using Ingenuity Pathway Analysis (IPA, Qiagen).

### Statistical analysis

All results were expressed as mean ± SEM. Statistical comparisons between the groups were made using two-way ANOVA with the Tukey–Kramer test. Differences between the experimental groups were considered statistically significant at a *p* < 0.05 level. *F*-values representing the ratio of variance of two independent variables and their interaction effect were also determined. Survival results were plotted as Kaplan–Meier survival curves, and significance was calculated using the Log-rank (Mantel-Cox) test. All statistical comparisons were performed using GraphPad Prism software version 9.

## Results

### Hypertension and age increase susceptibility to respiratory infection caused by *Francisella tularensis* LVS

**Figure 1:**
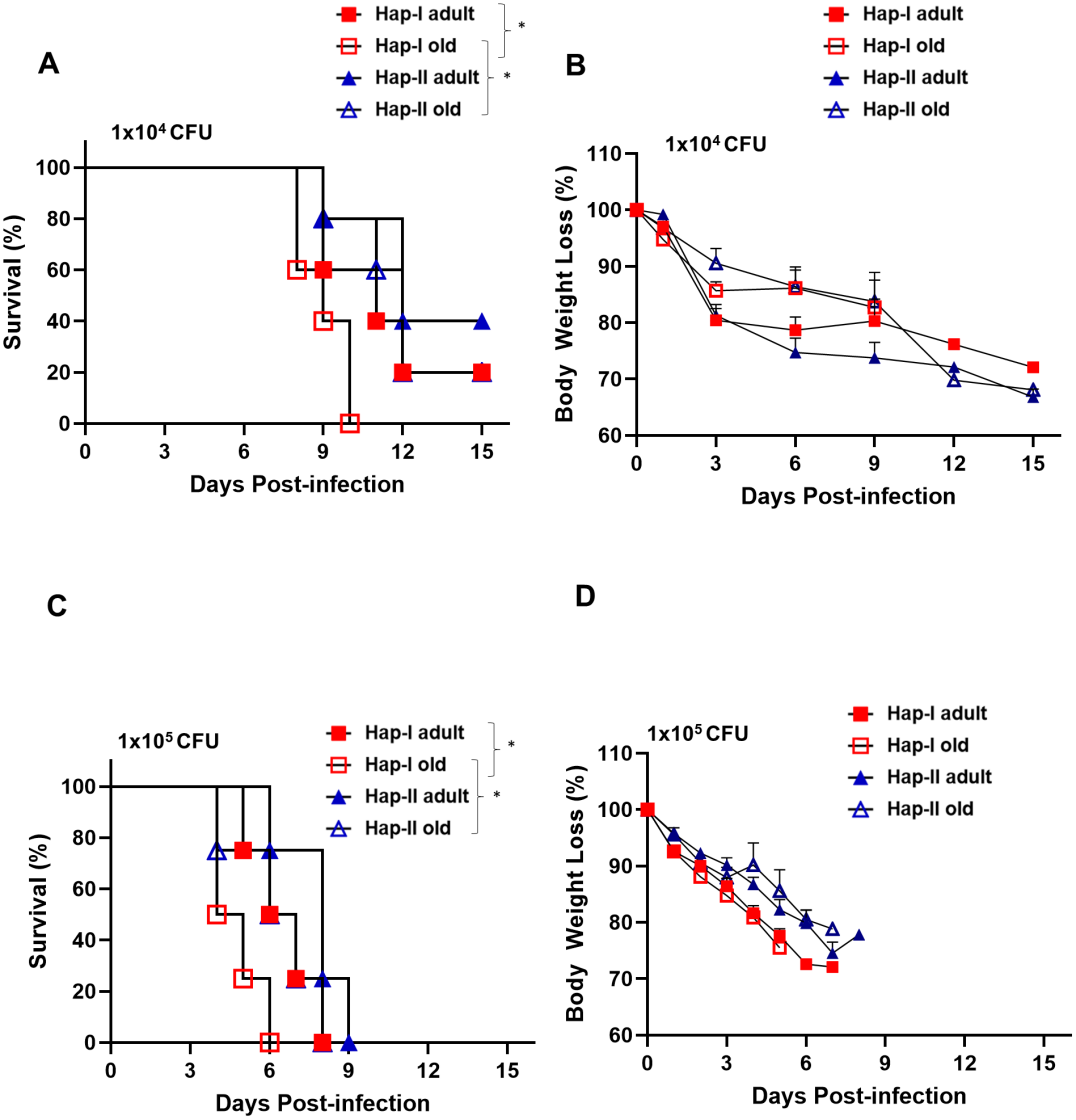
Susceptibility of Hap-I and Hap-II transgenic (TG) mice to *Francisella tularensis* LVS infection. Adult and old Hap-I (hypertensive) and Hap-II (normotensive-control) TG mice (*n* = 5 per group) were infected intranasally with 1 × LD_100_ (1 × 10^4^ CFU) **(A,B)** or 10 × LD_100_ (1 × 10^5^ CFU) **(C,D)** dose of *F. tularensis* LVS and were observed for morbidity and mortality for 15 days post-infection. Survival results are plotted as Kaplan-Meir survival curves **(A,C)** and the body weights are represented as percent initial body weight **(B,D)**. The survival data were analyzed using the Log-rank test. *p < 0.05.

The association of hypertension and old age with susceptibility to respiratory tularemia was determined using hypertensive Hap-I TG adult (12–16 weeks old) and old (72–80 weeks old) mice. Age-matched normotensive Hap-II TG adult and old mice were used as controls. Hap-I and Hap-II TG adult and old mice were infected intranasally with 1 × (1 × 10^4^ CFU) and 10× (1 × 10^5^ CFU) lethal doses of *F. tularensis* LVS and were observed for morbidity and mortality for 15 days post-infection. The infection doses selected were based on our several previous studies (Alqahtani et al., 2018; Ma et al., 2019; Marghani et al., 2021; Suresh et al., 2021). No differences between the survival proportions of Hap-II TG adult and old mice were observed, and 60 % of mice from both groups succumbed to infection with 1 × 10^4^ CFUs of *F. tularensis* LVS with a median survival time (MST) of 12 days. Eighty percent of the Hap-I TG adult mice died with an MST of 11 days. However, 100% of the Hap-I TG old mice succumbed to infection with a significantly lower (*p* < 0.05) MST of 9 days as compared to Hap-I adult (11 days) mice (Figure 1A). A significant difference (*p* < 0.05) in MST between Hap-I old (9 days) and Hap-II old (12 days) mice was also observed. Mice from all the groups lost their body weight, indicating that all mice received the infection (Figure 1B). At a higher infection dose of 1 × 10^5^ CFU, 100% mortality was observed in all groups of infected mice. All Hap-I TG old mice died by day six post-infection with a significantly lower (*p* < 0.05) MST of 4.5 days as compared to Hap-I TG adult mice (6.5 days). Hap-II TG adult and old mice succumbed to infection by day 8–9 post-infection (MST 6.5–8 days). As observed with the lower dose, a significant difference (*p* < 0.05) in MST between Hap-I old (4.5 days) and Hap-II old (6.5 days) mice was also observed. All infected mice lost their body weights, and their weight loss was more rapid than that observed for those infected with 1×10^4^ CFU of *F. tularensis* LVS (Figures 1C,D). Together, these results demonstrate that hypertensive (Hap-I) old mice are more susceptible to acute respiratory infection caused by *F. tularensis* than hypertensive adults and the normotensive (Hap-II) adult and old mice.

### Hypertension and age are associated with impaired clearance of bacteria during an acute respiratory infection caused by *Francisella tularensis* LVS

**Figure 2:**
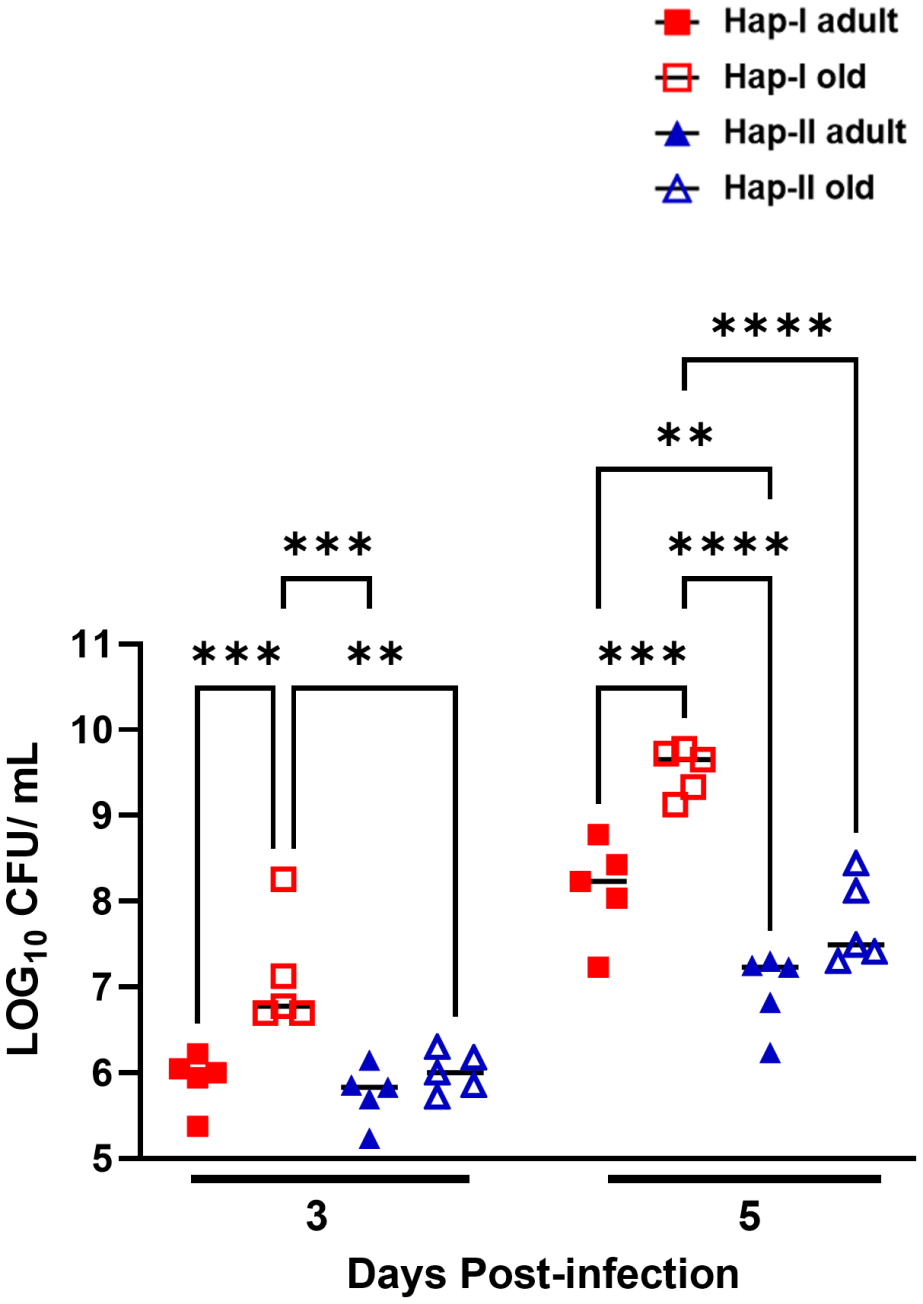
Bacterial load in the lungs of Hap-I and Hap-II mice. Adult and old Hap-I (hypertensive) and Hap-II (normotensive-control) TG mice (*n* = 4 per group) were infected intranasally with 1 × LD_100_ (1 × 10^4^ CFU) dose of *F. tularensis* LVS. The bacterial burdens were quantitated in the lungs on days three and five post-infection. Bacterial numbers are expressed as Log_10_ CFU/gram of tissue. The *p*-values were determined using two-way ANOVA. **p < 0.01; ***p < 0.001; ****p<0.0001.

It was observed that the hypertensive Hap-I TG old mice were more susceptible to *F. tularensis* LVS infection than their adult and normotensive counterparts. We next determined if the enhanced susceptibility of Hap-I old mice is associated with their reduced ability to clear bacteria from the site of infection. We infected age-matched Hap-I and Hap-II adult and old mice with 1 × 10^4^ CFUs of *F. tularensis* LVS intranasally. Mice were sacrificed on days 3 and 5 post-infection, and their lungs were collected aseptically. The homogenates of the lungs were prepared, diluted 10-fold, and plated on MH chocolate agar plates to quantitate the bacterial load. The bacterial load was significantly higher (*p* < 0.01) in the Hap-I old than in Hap-I adult and Hap-II old mice on day three post-infection. The bacterial load increased in all groups of mice by day five post-infection. However, similar to day three, significantly higher (*p* < 0.001) numbers of bacteria were recovered from the lungs of Hap-I old than Hap-I adult and Hap-II adult and old mice. Further, like day three, on day five post-infection, the bacterial burdens were also significantly higher (*p* < 0.01) in Hap-I adult than in Hap-II adult mice. However, no statistical differences were observed in the bacterial load between Hap-II adult and old mice on days three and five post-infection (Figure 2). Overall, a statistically significant interaction was observed between bacterial burden in Hap-I and Hap-II mice with age and *F. tularensis* LVS infection [*F* (3.32) = 3.644, *p* = 0.0229]. Collectively, these results demonstrate that a hypertensive phenotype alone or along with old age impaired the ability to control bacterial growth from the site of infection which ultimately results in enhanced susceptibility to infection and adverse outcomes.

### Hypertension and age are associated with severe organ damage during an acute respiratory infection caused by *Francisella tularensis*

**Figure 3:**
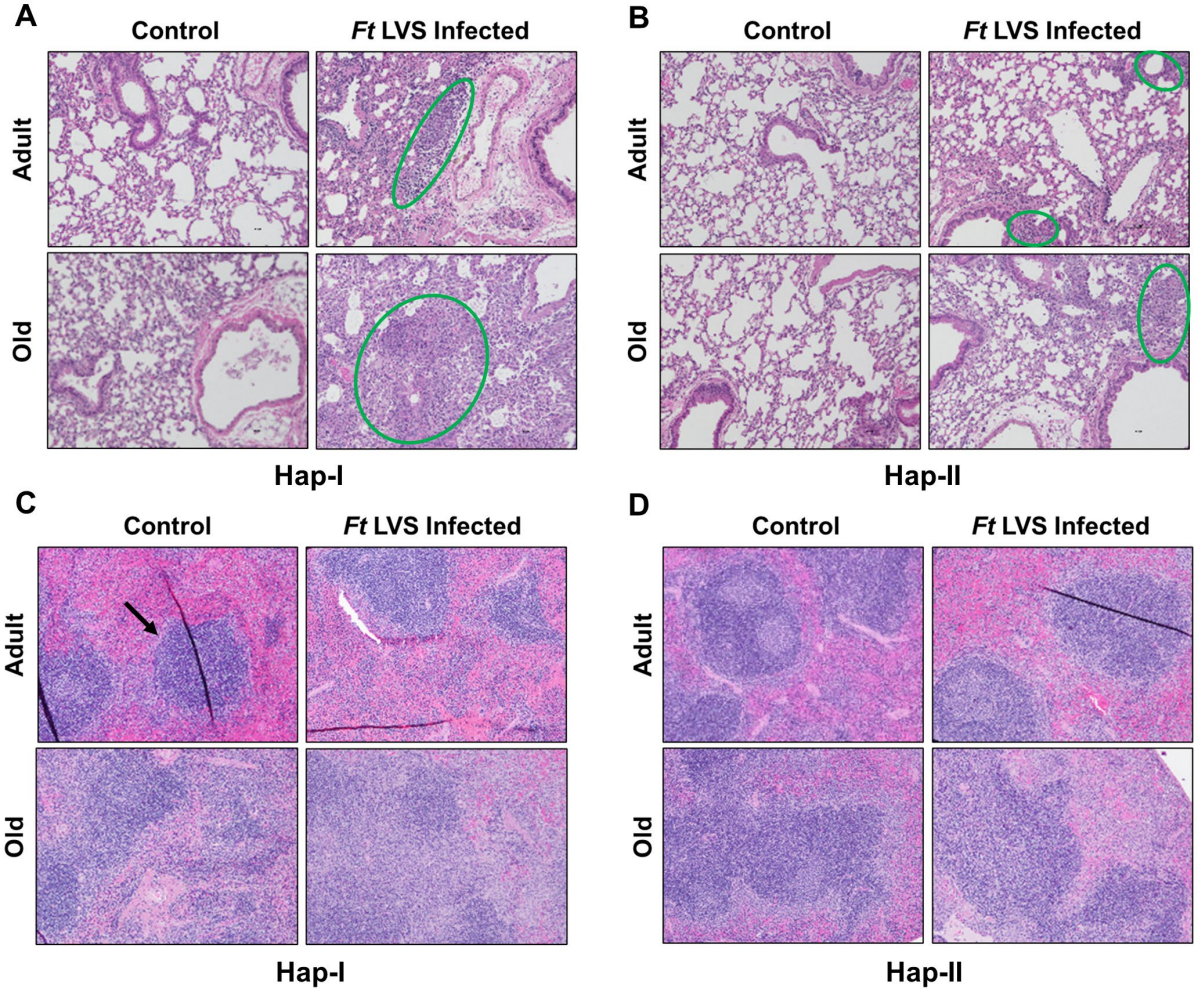
Histopathological lesions in the lungs and spleen of Hap-I and Hap-II mice. Adult and old Hap-I (hypertensive) and Hap-II (normotensive-control) TG mice (*n* = 4 per group) were infected intranasally with 1 × LD_100_ (1 × 10^4^ CFU) dose of *F. tularensis* (*Ft*) LVS. Lungs **(A,B)** and spleens **(C,D)** were excised on day five post-infection, preserved in 10% formalin, paraffin-embedded, sliced into 3 μ thin sections and stained with Hematoxylin & Eosin. Stained sections were observed for histopathological lesions under a light microscope (Magnification 20×). The green circles **(A,B)** show alveolar and peribronchial inflammatory foci. The black arrow **(C)** shows normal splenic architecture.

It was next determined if the higher bacterial load in the lungs of Hap-I TG adult and old mice is also associated with lung and spleen damage. Lungs and spleen from Hap-I and Hap-II adult and old mice infected intranasally with 1 × 10^4^ CFUs of LVS were collected on day five post-infection, embedded in paraffin, sectioned, and stained with H&E to observe histopathological changes. No pathological changes were observed in the lungs of control uninfected Hap-I adult and old mice, indicating that the hypertensive phenotype *perse* does not cause any effect in the lungs of Hap-I adult or old mice. The lungs of Hap-I adult mice infected with *F. tularensis* LVS showed patchy alveolar and peribronchial inflammatory foci. However, in infected Hap-I old mice, these foci of inflammation were more severe and developed into necrotizing pneumonia at several places (Figure 3A). Similar to uninfected Hap-I mice, lungs of uninfected Hap-II adult and old mice did not reveal any pathological lesions. Only mild and small patchy inflammatory foci were observed in the lungs of infected Hap-II adult and old mice, while most of the lung parenchyma was free of inflammatory lesions (Figure 3B).

Unlike lungs, the spleens of uninfected Hap-I old but not Hap-I adult mice revealed inflammatory, proliferative responses within the germinal centers. The lesions in the spleens of infected Hap-I adult mice revealed disrupted splenic red pulp. In *F. tularensis* LVS-infected Hap-I old mice, the lesions in the spleen presented with coalescing areas of neutrophilic to pyogranulomatous necrotic splenitis that resulted in complete disruption of splenic architecture (Figure 3C). The spleens of Hap-II adult mice showed normal architecture with minimal evidence of inflammatory response on day five post-infection, while a proliferative response was observed in the spleen of Hap-II old mice (Figure 3D). Taken together, results shown in Figures 1–3 demonstrate that failure to control the bacterial load by Hap-I old mice results in a hyperinflammatory response in the lungs and spleen causing severe organ damage, enhanced susceptibility, and early death when infected with *F. tularensis* LVS.

### Increased levels of pro-inflammatory cytokines are observed in Hap-I old mice infected with *Francisella tularensis* LVS

**Figure 4:**
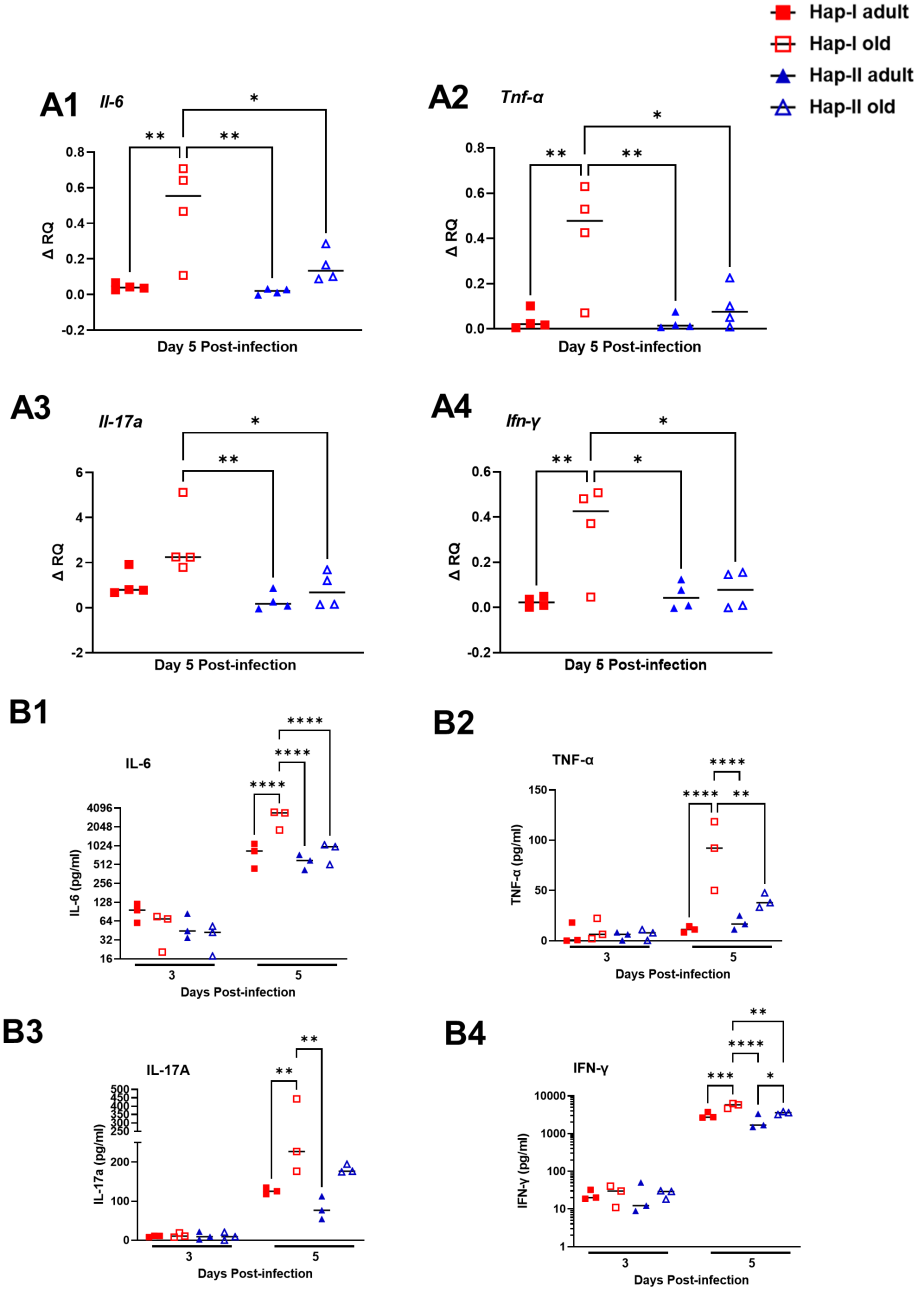
Expression of inflammatory cytokines in the lungs of *F. tularensis* LVS-infected Hap-I and Hap-II TG mice. Adult and old Hap-I (hypertensive) and Hap-II (normotensive-control) TG mice were infected intranasally with 1 × 10^4^ CFU of *F. tularensis* LVS. QRT-PCR performed using RNA extracted from the lungs (*n* = 4 per group) on day five post-infection **(A1–A4)**. The serum levels of the indicated cytokines (*n* = 3 mice per group) were assayed by ELISA days three and five post-infection with *F. tularensis* LVS **(B1–B4)**. The *p*-values were determined using two-way ANOVA. *p < 0.05, **p < 0.01, ***p < 0.001; ****p < 0.0001.

Initially, we determined the basal expression levels of *il-6, Tnf-α*, *Il-17*a and *ifn-γ* transcripts in uninfected Hap-I and Hap-II, adult and old mice by qRT-PCR. No differences were observed in the transcript levels of these cytokines (Supplementary Figure S1). We further determined the transcripts level of these cytokines on day five post-infection. As shown in Figure 4, the transcript levels of all the cytokines tested were significantly increased in old Hap-I mice as compared to adult Hap-I (except *il-17a*) as well as their Hap-II counterparts on day five post-infection (Figures 4A1–A4). We further confirmed the results of qRT-PCR by determining the serum cytokine levels on day three and day five post-infection in these mice. No significant changes were observed in cytokine levels on day three post-infection in adult and old, Hap-I or Hap-II mice. However, as observed for the transcript levels, all the cytokine levels were significantly higher in old Hap-I mice as compared to their Hap-II counterparts on day five post-infection (Figures 4B1–B4). Overall, these results indicate that *F. tularensis* LVS infection leads to a haplotype and age-dependent augmentation of an inflammatory response which may cause increased susceptibility to infection and organ damage in *hAT1R* Hap-I TG mice.

### *Francisella tularensis* LVS infection results in enhanced expression of RAS molecules in aged TG mice

**Figure 5:**
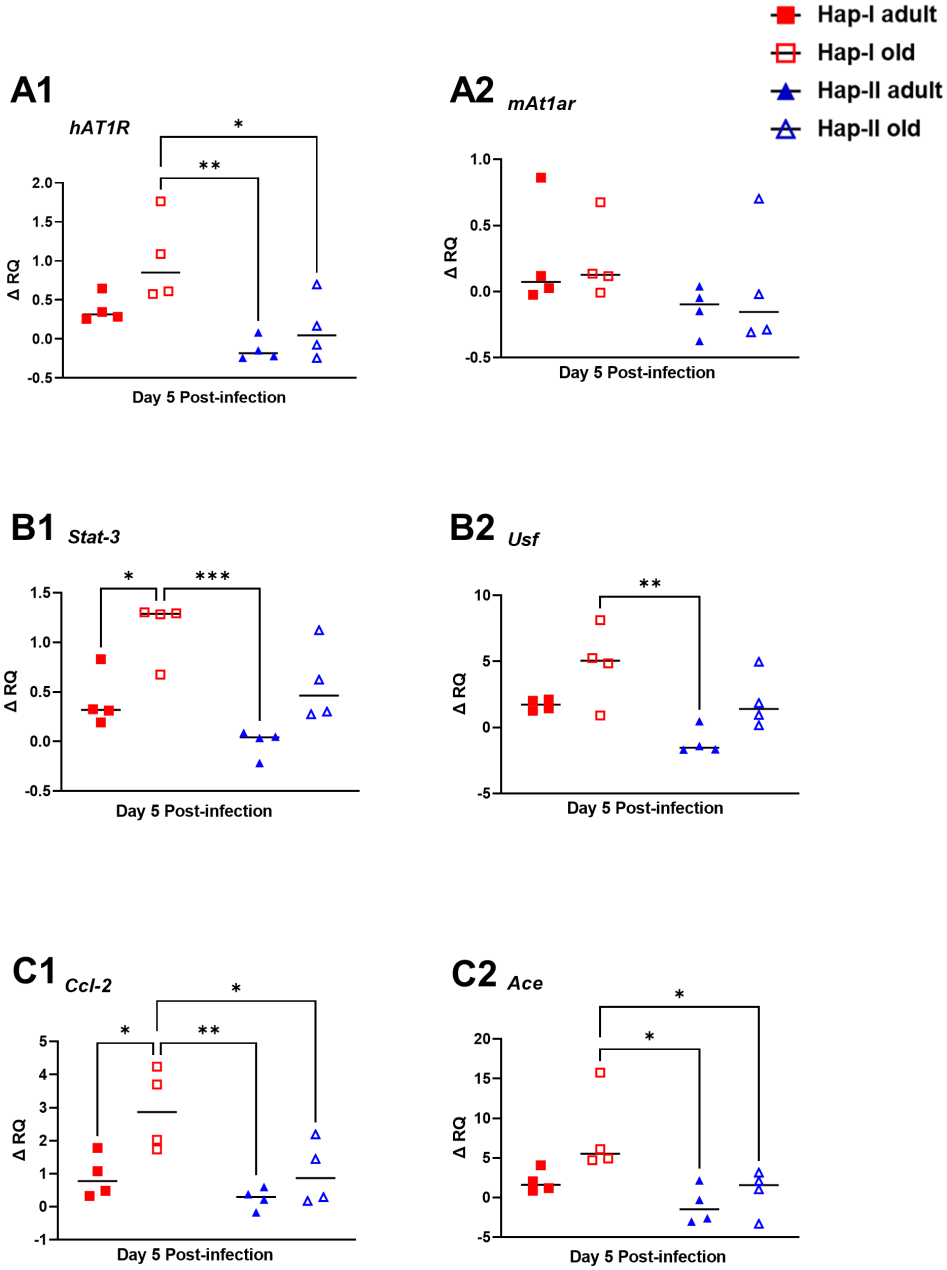
Expression of molecules involved in RAS signaling pathway in the lungs of *F. tularensis* LVS infected Hap-I and Hap-II mice. Adult and old Hap-I (hypertensive) and Hap-II (normotensive-control) TG mice (*n* = 4 per group/time) were infected intranasally with 1 × 10^4^ CFU of *F. tularensis* LVS. RNA isolated from lungs on day five post-infection was used for quantitation of expression of human **(A1)** and mouse **(A2)** *At12* genes, and other indicated molecules involved in RAS signaling pathway by qRT-PCR **(B1,B2,C1,C2)**. Relative quantification of transcripts (ΔRQ) was done by subtracting the change in transcript levels of each control subject post-*F. tularensis* LVS infection. The *p*-values were determined using two-way ANOVA. *p < 0.05, **p < 0.01; ***p < 0.001.

Previously we have shown age and diet-dependent regulation of *hAT1R* gene expression in Hap-I and Hap-II *hAT1R* TG mice (Jain et al., 2018a,b). Therefore, in the present study, we examined the expression of the *hAT1R* gene following infection with *F. tularensis* LVS in adult and old hAT1R TG mice. RNA isolated from the lungs of *F. tularensis* LVS-infected old Hap-I mice showed significantly high *hAT1R* expression (*p* < 0.01) as compared to the adult and old Hap-II mice (Figure 5A1). However, the expression of endogenous mouse *At1ar* (*mAt1ar*) gene remained unaltered in both haplotypes (Figure 5A2). Higher levels of hAT1R in old Hap-I mice suggested a haplotype-specific increase in RAS, driven by *F. tularensis* LVS infection.

Additionally, published data from our earlier studies suggest that age and diet potentiate transcriptional milieu in Hap-I TG mice and upregulate *Usf* and *Stat3* genes, which bind strongly to promoter of the *hAT1R* gene (Jain et al., 2018a,b). To investigate the expression of *Usf* and *Stat3* in *F. tularensis* LVS-infected adult and old Hap-I TG mice, we quantified them in the lungs by qRT-PCR. Our results indicate a highly significant age-dependent increase in *Stat3* and *Usf* expression in Hap-I hypertensive as compared to Hap-II mice (Figures 5B1, B2). These results suggest that infection in aged subjects leads to an increased expression of *Stat3 and Usf* transcription factors which over-drive the transcription of *hAT1R* gene, specifically in Hap-I TG mice, and therefore may cause RAS augmentation.

Angiotensin convertase enzyme (ACE) catalyzes the conversion of angiotensinogen into a vasoactive octapeptide AngII. Overexpression of ACE, thus, leads to vasoconstriction and an increase in blood pressure, causing hypertension. Conversely, CCL2 is an inflammatory and immunomodulatory cytokine involved in disease pathogenesis. Therefore, we analyzed the expression of *Ace* and *Ccl2* genes in the *F. tularensis* LVS-infected lungs of adult and old Hap-I and Hap-II mice. The qRT PCR results show a significant increase in *Ace* and *Ccl2* gene expression in Hap-I old TG mice as compared to the adult or old Hap-II TG animals (*p* < 0.01). This expression is age dependent as we did not observe any significant difference in either *Ace* or *Ccl2* gene expression in adult mice (Figures 5C1, C2). These results indicate the role of *Ace* and *Ccl2* gene overexpression in the activation of the RAS pathway following infection with *F. tularensis* LVS.

### Transcriptional analysis of lung tissues from Hap-I mice infected with *Francisella tularensis* LVS reveals modulations of hypertension and infection-associated canonical signaling pathways

**Figure 6:**
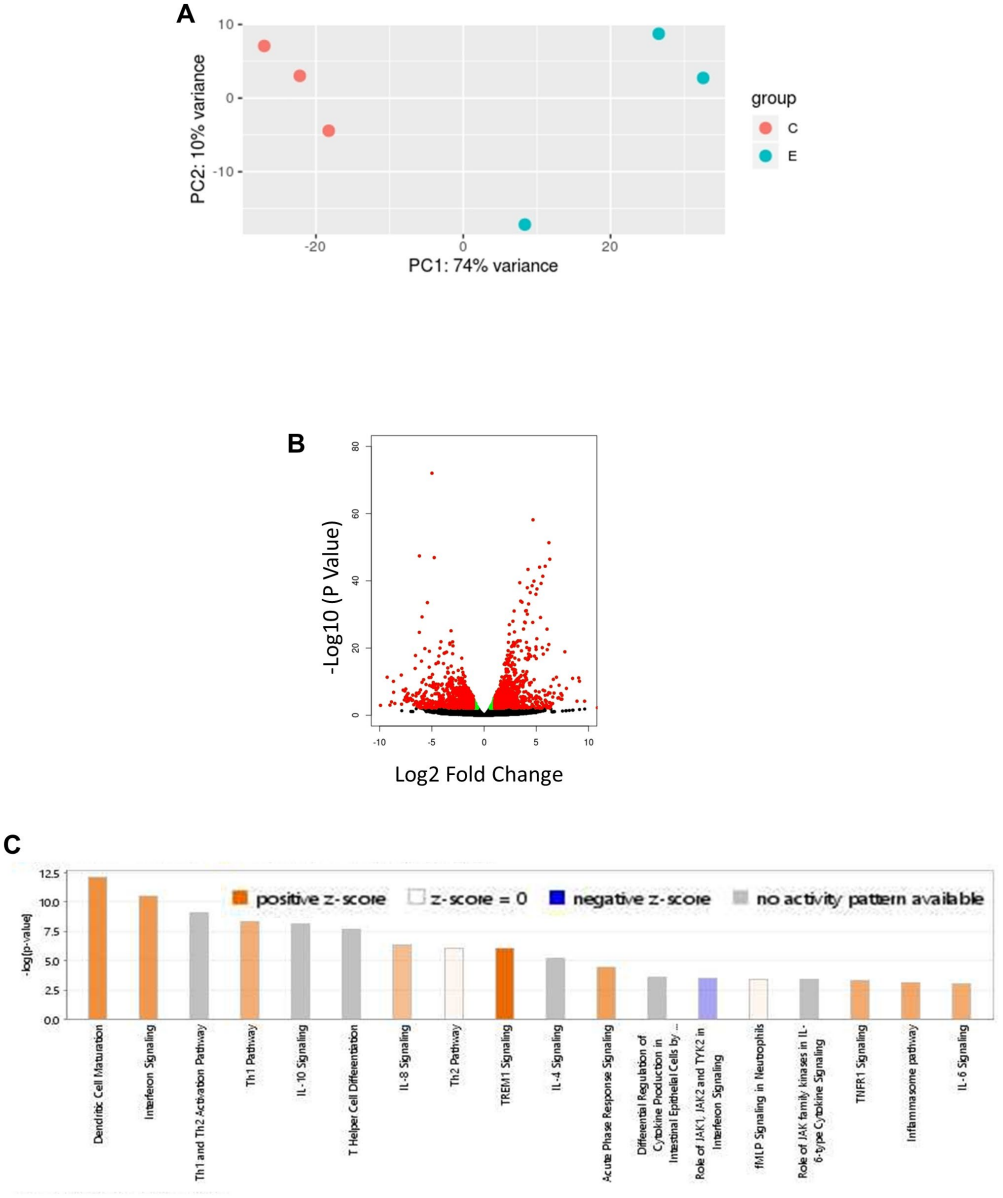
Bioinformatic analysis of transcriptomic data obtained from lungs of *F. tularensis* LVS-infected old Hap-I mice as compared to their age matched vehicle control group. PCA plot shows distinct isolation of uninfected control and *F. tularensis* LVS-infected Hap-I old mice **(A)**. The volcano plot shows a significant alteration of >1,400 genes with *p* < 0.005 **(B)**. Significant canonical pathways related to immune responses, including interferon signaling **(C)**. *n* = 3, *p* < 0.005.

Age-related hypertension and infection may alter the expression of genes in canonical pathways associated with blood pressure regulation and inflammation. Therefore, we next investigated if the altered profile of genes modulating RAS and IFN-γ pathways as the major regulatory mechanisms, respectively, influencing blood pressure and inflammation. RNA sequencing was performed on lung RNA from *F. tularensis* LVS-infected old Hap-I and age-matched uninfected control animals. The gene expression profile was analyzed by IPA software from Qiagen. As seen in the PCA plot, the results from RNA sequencing analysis indicate a clear segregation of gene expression profiles of uninfected control and *F. tularensis* LVS-infected Hap-I mice (Figure 6A). More than 1,400 genes were significantly altered in old Hap-I mice infected with *F. tularensis* LVS as shown in the Volcano plot (Figure 6B). Almost 50 percent of genes were upregulated. Canonical pathways associated with infection, including cytokine signaling and immune cell activation were significantly altered in hypertensive haplotype Hap-I (Figure 6C). Further, the results from gene expression analysis showed a higher level of cytokines genes including *Il-1, Il-6, Il-17, Ifn-γ, Tnf-α* and their receptors post-*F. tularensis* LVS-infection (Table 1A); a significant upregulation of genes involved in RAS pathway including *Ccl2, Nfkb, p38 Mapk, Ras, Stat1, Stat3 and Tnf-α* (Table 1B); and an upregulated expression of IFN-γ pathway genes such as *Bak, Bax, Ifit, Ifn-γ, Irf, Isg, Oas1, Psmb8, Ptpn2, Stat* and *Tap1* (Table 1C). The RNA-Seq data are available in the GEO database under accession number GSE186016. Results from the RNA-seq gene expression analysis were corroborated by qRT-PCR analysis for the genes involved in the RAS pathway and pro-inflammatory cytokines of the IFN-γ pathway.

**Table 1:** Expression of RAS and IFN-γ pathway specific molecules as analyzed by IPA (Qiagen) in the lungs of aged hAT1R TG mice post-*Francisella tularensis* LVS infection.

A		B		C	
Pro-inflammatory cytokines	RAS pathway molecules	IFN-γ signaling molecules	Gene symbol	Expr log ratio	Gene symbol	Expr log ratio	Gene symbol	Expr log ratio
*Il17ra*	0.598	*Agtr1*	−1.657	*Bak1*	2.325
*Il17a*	6.355	*Ccl2*	2.141	*Bax*	0.997
*Il6*	4.792	*Nfkb*	1.190	*Ifit1*	2.261
*Il1rl1*	2.191	*P38 Mapk*	2.580	*Ifit3*	3.419
*Il1b*	0.654	*Pi3K*	1.540	*Ifitm3*	2.018
*Il1a*	2.797	*Ptpn6*	1.191	*Ifng*	6.046
*Ifng*	6.046	*Ras*	2.900	*Irf1*	2.586
*Ifngr2*	0.259	*Stat1*	2.948	*Irf9*	1.116
*Il1r2*	3.473	*Stat3*	0.919	*Isg15*	3.977
*Tnf*	2.915	*Tnf*	2.915	*Oas1*	3.218
*Tnfrsf12a*	1.611			*Psmb8*	2.810
*Tnfrsf21*	0.960			*Ptpn2*	1.320
				*Socs1*	4.046
				*Stat1*	2.948
				*Stat2*	2.304
				*Tap1*	3.503

## Discussion

Old age as a contributing factor for increased susceptibility or the severity of the infection is not new. It has been demonstrated that age-related mortality is more commonly associated with pathogens capable of causing both acute and chronic infections (Kline and Bowdish, 2016). The increased susceptibility to infection in aged people is mostly associated with a decline in cell-mediated immune responses, changes in the structure and functions of the organs, especially the lungs, and degenerative diseases (Boe et al., 2017). Additionally, comorbidities such as hypertension, obesity, and diabetes also increase susceptibility to infectious diseases (Vasto et al., 2007; Shaw et al., 2013). Hypertension is the most prevalent disease and a leading cause of vascular disorders and deaths worldwide (Mills et al., 2020). Several parasitic, viral, and bacterial infectious diseases have been linked to hypertension. Higher susceptibility to malaria, COVID-19, HIV, dengue, herpesvirus, cytomegalovirus, *Chlamydia* and *Helicobacter* infections have been reported in individuals with hypertension (Cool et al., 2003; Sotiropoulos et al., 2006; Cheng et al., 2009; Mehta and Hotez, 2016; Fahme et al., 2018; Etyang et al., 2019). On the other hand, chronic bacterial periodontal diseases cause hypertension by increasing both systolic and diastolic blood pressures (Desvarieux et al., 2010). Chronic inflammation caused by hypertension has been proposed to be one of the major factors responsible for increased susceptibility and higher mortality due to these infectious diseases (Tseng et al., 2020). The complications related to hypertension get further aggravated in aged subjects. However, a lack of effective models to study the molecular mechanisms associated with hypertension and its contribution to enhanced susceptibility to infectious diseases during old age remain elusive. Previously, we generated TG mouse models of chronic hypertension exhibiting age and diet-related upregulation of RAS and associated renovascular disorders. The two lines of TG mice have variants of the human *AT1R* gene represented by haplotype-I (Hap-I, hypertensive genotype) and haplotype-II (Hap-II, normotensive genotype). In this study, we used pneumonic tularemia as an infectious disease model and old Hap-I and Hap-II TG mice to study the impact of hypertension and old age on susceptibility and immunopathogenesis of *F. tularensis* infection.

Immuosenescence, the changes that occur to immune system with aging, and inflammaging, the age-related increase of pro-inflammatory cytokine levels in blood and tissues are the hallmarks of the immune dysfunction associated with old age. Both immuosenescence and inflammaging diminish resistance to fight infection in old age. Immune dysfunctions associated with immuosenescence include defects in T and B cell development resulting in impaired innate and adaptive immune responses to pathogens and immunogens (Cohen et al., 2003; Arnold et al., 2011; Kogut et al., 2012). The number of neutrophils, monocytes and DCs as a result of immuosenescence remain unchanged or mildly decreased in aged individuals indicating that the increased basal inflammation associated with inflammaging is not due to increased cellularity (Chatta et al., 1994; Jing et al., 2009; Seidler et al., 2010). Impaired chemotaxis of neutrophils which accounts for both reduced migration as well as egression from the sites of inflammation, diminished capacity to phagocytose and kill phagocytosed bacteria by neutrophils are the most common features of immuosenescence (Wenisch et al., 2000; Butcher et al., 2001; Gomez et al., 2007). NK cell infiltration in lungs, cytotoxicity, IFN-γ production, and the effector functions of macrophages and DCs are also impaired with age (Moretto et al., 2008; Beli et al., 2011; Hearps et al., 2012).

A previous study conducted in old (20–24 months) C57BL/6 mice using *F. tularensis* LVS as a respiratory infection model demonstrated that old age alone does not impair the ability to clear bacteria from the lungs. In fact, old mice clear *Francisella* more efficiently than young mice. Although the histopathological lesions are more extensive in the lungs of old than young mice, no differences were observed in the survival of old and young mice (Mares et al., 2010a,b). Similar results have been reported for infections with several intracellular pathogens in young and old mice (Emmerling et al., 1979; Turner et al., 2002; Ehrchen et al., 2004), indicating that old age associated immuosenescence alone does not contribute to enhanced susceptibility to *Francisella* infection. The results obtained in the present study in normotensive Hap-II old mice agree with these results. No significant differences in bacterial burdens and survival were observed between Hap-II adult and Hap-II-old mice. However, the histopathological lesions in the lungs and spleen were more severe in Hap-II old than in the respective Hap-II adult mice. Contrary to this, hypertensive old Hap-I mice had a significantly higher bacterial load in the lungs than in their younger hypertensive or adult and old normotensive counterparts. This increased bacterial load in the lungs resulted in severe organ damage, leading to significantly increased mortality in old Hap-I mice. These results clearly demonstrate that old age and hypertension together, rather than old age alone or a hypertensive phenotype in young adults, increases the susceptibility and severity of respiratory tularemia caused by *F. tularensis.*

The inflammaging-associated immune dysfunction include elevated basal levels of IL-6, which transforms monocytes into antigen presenting cells to produce IL-1β and IL-23. Both IL-1β and IL-23 amplify the production of IL-17A, which is responsible for causing end-organ damage and hypertension. IL-17A has been reported to be a key pro-inflammatory cytokine associated with hypertension in individuals suffering from autoimmune disorders such as systemic lupus erythematosus (SLE) and Crohn’s disease (Toussirot, 2012; Lozovoy et al., 2014). Furthermore, IL-1β produced by the myeloid cells is also associated with exacerbation of hypertension (Alexander et al., 2019). In addition to these proinflammatory cytokines, the immune cells further exacerbate hypertension by releasing reactive oxygen species (ROS), and metalloproteinases (Madhur et al., 2010). Monocytes and macrophages, the cells primarily involved in innate immune response, also augment the development of hypertension. Mice that lack macrophage-colony stimulating factor are protected from the rise in blood pressure (De Ciuceis et al., 2005). Furthermore, monocyte-derived derived DCs from hypertensive mice produce increased amounts of ROS, IL-1β, TNF-α, IL-6, and IL-23 to promote hypertension and end-organ damage. The production of these inflammatory mediators by DCs in hypertensive individuals also skew the CD8^+^ T cells to produce IFN-γ and IL-17 (Kirabo et al., 2014). B and T lymphocytes cells are also linked to hypertension, as RAG1 and SCID mice that lack both the T and B cells, and BAFF-R mice, that lack mature B cells are protected from hypertension (Guzik et al., 2007; Crowley et al., 2010; Chan et al., 2015). These studies highlight the importance of inflammaging in augmenting hypertension and the associated end-organ damage.

A dysregulated host response associated with elevated levels of pro-inflammatory cytokines and chemokines is a hallmark of the severity of *Francisella* infection, progression to sepsis and death (Mares et al., 2008; Sharma et al., 2011; Spencer et al., 2021). Elevated levels of pro-inflammatory cytokines TNF-α, IL-6 and chemokines are the markers of sepsis. It has been demonstrated in previous studies that both young and old mice can mount immune responses of similar magnitude to several intracellular pathogens, which potentially accounts for the lack of differences in susceptibility to infections as a function of old age (Emmerling et al., 1979; Turner et al., 2002; Ehrchen et al., 2004). It has been reported that the magnitude of the pro-inflammatory cytokine response is of a lower magnitude in old mice infected with *F. tularensis* LVS than in young mice (Mares et al., 2010a). This was proposed to be due to the early clearance of bacteria by the old mice. Since we observed a higher bacterial load and severe histopathological lesions in *F. tularensis* LVS-infected Hap-I old mice, we investigated whether these differences were due to an altered immune environment in the lungs of Hap-I hypertensive old mice.

The RNA sequencing analysis of the lung tissues of Hap-I old mice revealed the differential expression of several pro-inflammatory cytokine genes. The *Tnf-α*, *Il-6*, *Ifn-γ,* and *l1-17a* were significantly upregulated in the lungs of Hap-I old mice as compared to the Hap-I adult, Hap-II adult, and Hap-II old mice. The upregulated expression of the cytokine genes as well as protein levels are akin to the cytokine storm observed in the later stages of *Francisella* infection. It has been reported that moribund mice express higher levels of these cytokines resulting from excessive bacterial replication and severe organ damage and correlate with disease severity, sepsis, and mortality. It is probable that inflammaging in Hap-I old mice may further aggravate inflammation and organ damage due to *Francisella* infection. Upregulation of these inflammatory markers further support our preceding results and establish that hypertension and age, as observed in Hap-I old mice, will increase susceptibility to respiratory tularemia caused by *F. tularensis*. In addition to these immunological factors, oxidative stress and mitochondrial dysfunction associated with age also contributes to age-related hypertension (Griendling et al., 2021). Necroptosis caused by *Francisella* results in severe mitochondrial damage, increased mitochondrial oncosis, and oxidative stress, leading to the leakage of mitochondrial contents in the lungs of infected mice. The recognition of released mitochondrial contents results in elevated levels of pro-inflammatory cytokines, contributing to sepsis-like conditions observed during *Francisella* infection (Singh et al., 2017). The results from this study on day three post-infection demonstrate that despite significant, but small differences in bacterial loads between Hap-I adult and old mice in lungs, the cytokine levels remain more or less the same. These data support the notion that changes in pro-inflammatory cytokine levels in Hap-I old mice are not on account of the differences in the bacterial load observed on day five post-infection, but probably are caused by the immunoregulatory role of the AT1R gene. On the other hand, lower levels of these cytokines and chemokines in Hap-I adult, Hap-II adult, and old mice indicate that hypertensive phenotype or old age alone can tip the balance of this pro-inflammatory response to a beneficial side leading to bacterial clearance and enhanced survival.

Multiple studies have shown the activation of AT1R signaling in the process of aging (Benigni et al., 2009; Capettini et al., 2012; Harvey et al., 2016). Human AT1R expression significantly increases in Hap-I mice with age. Findings from current studies show that *F. tularensis* LVS infection results in a substantial increase in human *AT1R* expression in old Hap-I subjects as compared to their old Hap-II counterparts. However, the mouse *At1r* does not show a significant age-dependent shift post-*F. tularensis* LVS infection. Overexpression of *hAT1R*, in turn, significantly activates RAS signaling in Hap-I animals, thereby assisting an infection-associated phenotype. ACE directly influences various aspects of the immune response and is a chief regulator of the RAS pathway and cleaves angiotensin I into the vasoconstrictor angiotensin II. It has been shown that an ACE inhibitor reduces the bactericidal activity of human neutrophils *in vitro* and impairs mouse neutrophil activity *in vivo* (Cao et al., 2021). Besides regulatory molecules, a large number of chemokines also play a major role in hypertension and associated organ damage (Rudemiller and Crowley, 2017). CCL2 is a major chemokine that guides phagocyte infiltration to the sites of inflammation *via* chemokine receptor ligation (Fuentes et al., 1995; Johnston et al., 1999; Charo and Ransohoff, 2006). Additionally, the circulating levels of CCL2 are upregulated in patients with hypertension and correlate with the degree of hypertension-associated organ damage in humans (Tucci et al., 2006). The higher expression of *Ace* and *Ccl2* in *F. tularensis* LVS-infected old Hap-I mice from our study (Figures 5C1,C2) further supports these findings.

Recent studies have revealed global and tissue-specific transcriptomic alterations related to aging and infections (Lee et al., 2002; Rodwell et al., 2004; Alfego et al., 2018; Suresh et al., 2021). However, these studies do not provide molecular insight into hypertension. Therefore, by exploiting *hAT1R* overexpressing TG animals, it is possible to investigate further the underlying mechanisms and essential molecules associated with hypertension and vulnerability to infection. Some of these age and infection-related molecules may be involved in our haplotype-specific regulation of canonical pathways involved in blood pressure regulation and inflammation. Previous studies from our group have shown that the *hAT1R* promoter interacts with some trans-regulatory elements, including USF2 and STAT3. Stronger binding of these transcription factors to the *hAT1R* promoter in Hap-I leads to higher expression of the *hAT1R* gene and, thus, upregulation of the RAS pathway. Earlier, we have shown a diet and age-dependent upregulation of *Usf* and *Stat3*, which is associated with an increase in *hAT1R* expression and increased blood pressure in Hap-I animals (Jain et al., 2018a,b). This study shows a significant increase in the expression of *Usf* and *Stat3* transcription factors post-*F. tularensis* LVS infection in the lungs of Hap-I TG mice. A rise in these transcription factors suggests that the Hap-I mice would have been experiencing high BP due to the upregulation of *hAT1R* gene expression and RAS pathway, thus resulting in increased mortality following *F. tularensis* LVS infection.

**Figure 7:**
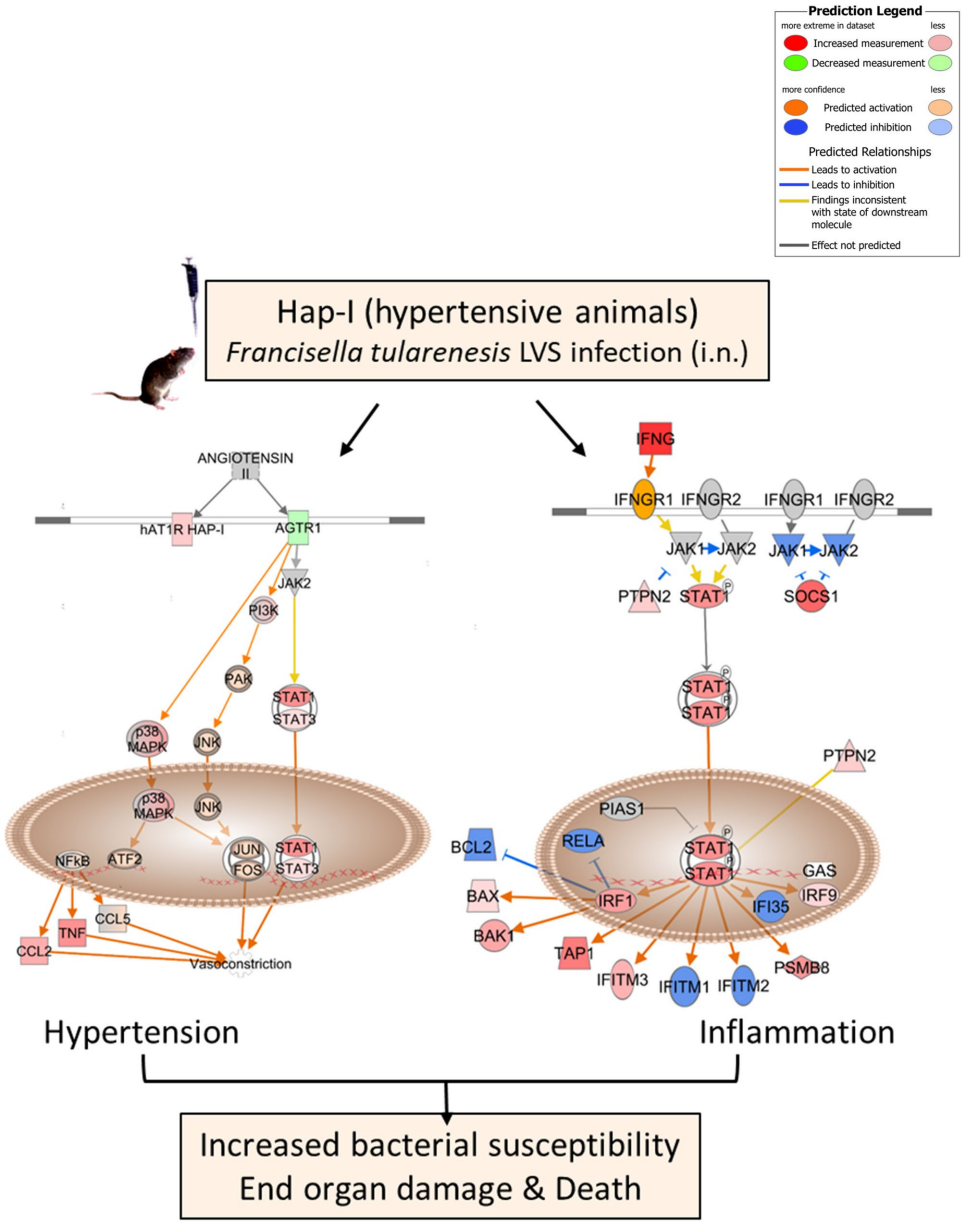
Potential mechanisms leading to increased susceptibility and end-organ damage in old Hap-I TG animals post-*F. tularensis* LVS infection. A schematic representation depicting upregulated expression of hypertension associated RAS and IFN-γ pathway mediated by overexpression of *hAT1R* gene in Hap-I hypertensive mice. The model was generated from the gene expression data obtained by RNA-sequencing utilizing IPA bioinformatic software (Qiagen).

Our results demonstrate an additive effect of aging and infection on inflammation and organ damage in a RAS-upregulated, chronically hypertensive mouse model. In the lungs, the Ang II-mediated classic RAS pathway regulates cell proliferation, hypoxia, angiogenesis, and several immune inflammatory responses, contributing to lung injury and pulmonary diseases, including those observed in the recent COVID-19 infection. These effects are exacerbated with aging (Monteonofrio et al., 2021). RAS inhibitors, primarily ACE and AT1R blockers, are critical in attenuating inflammation, vasoconstriction, and oxidative stress by reducing the availability and activity of vasoconstrictor Ang II. Previous studies suggest both beneficial and adverse effects of different RAS inhibitors on morbidity, mortality, and disease severity in subjects treated for infection (Tsampasian et al., 2022). As summarized in Figure 7, our results demonstrate that respiratory infection with *F. tularensis* LVS in old, hypertensive Hap-I mice results in upregulated expression of the hypertension-associated RAS pathway components, mediated by the *hAT1R* gene. Furthermore, upregulated expression of genes in the IFN-γ pathway *via* the gamma interferon activation site elements may contribute to the hyperinflammatory response and severe tularemia in old, hypertensive mice. The activation of these pathways eventually results in a hyperinflammatory response associated with enhanced organ damage and death of *F. tularensis*-infected old Hap-I hypertensive mice. Transcriptomic data from the lungs of old hypertensive animals provide us with novel gene targets associated with RAS and IFN-γ pathways to further explore the interaction of signaling mechanisms involved in aged hypertensive individuals in response to infection with other infections.

## Conclusion

Our results show that compared to the normotensive Hap-II mice expressing human AT1R, the hypertensive, transgenic Hap-I mice exhibit enhanced susceptibility to *F. tularensis* infection. This study demonstrates that severe organ damage and mortality are associated with aging in Hap-I mice due to an environment favoring the upregulation of components of the RAS signaling pathway following acute infection with *F. tularensis* LVS. Collectively, these findings pave the way for further studies investigating the association between age, hypertension, and other infections using a unique transgenic mouse model of chronic hypertension.

The impact of key components of the RAS pathway on proinflammatory cytokine signaling and the outcome of a bacterial infection is not well established. Our study, for the first time, addresses the association of the RAS pathway with hypertension, aging, and increased susceptibility to respiratory tularemia. Whether such an association is unique to *F. tularensis* infection or whether a similar response will be observed in our old, hypertensive Hap-I mouse model with other pathogens will remain the focus of our future investigations. It would also be of interest to evaluate the effect of various inhibitors on RAS signaling, immune response, and organ impairment in our mouse model in response to infection with other microbial pathogens.

## Data availability statement

The data presented in the study are deposited in the GEO database under accession number GSE186016.

## Ethics statement

Ethical review and approval was not required for the animal study because all animal procedures were conducted in accordance with the Institutional Animal Care and Use Committee (IACUC) guidelines approved by New York Medical College.

## Author contributions

HK, MA, ST, and SP carried out the experiments. HK, SJ, and CB analyzed the data. SJ and CB conceived and coordinated the research and drafted the manuscript. All authors contributed to the article and approved the submitted version.

## Funding

This research was funded by National Institutes of Health grant R01HL146628 to SJ.

## Conflict of interest

The authors declare that the research was conducted in the absence of any commercial or financial relationships that could be construed as a potential conflict of interest.

## Publisher’s note

All claims expressed in this article are solely those of the authors and do not necessarily represent those of their affiliated organizations, or those of the publisher, the editors and the reviewers. Any product that may be evaluated in this article, or claim that may be made by its manufacturer, is not guaranteed or endorsed by the publisher.
