# Supplementary figures and images for: Chronically hypertensive transgenic mice expressing human AT1R haplotype-I exhibit increased susceptibility to Francisella tularensis
Source: Front Microbiol. 2023 May 17;14:1173577. doi: 10.3389/fmicb.2023.1173577 (PMC10229887; doi:10.3389/fmicb.2023.1173577)

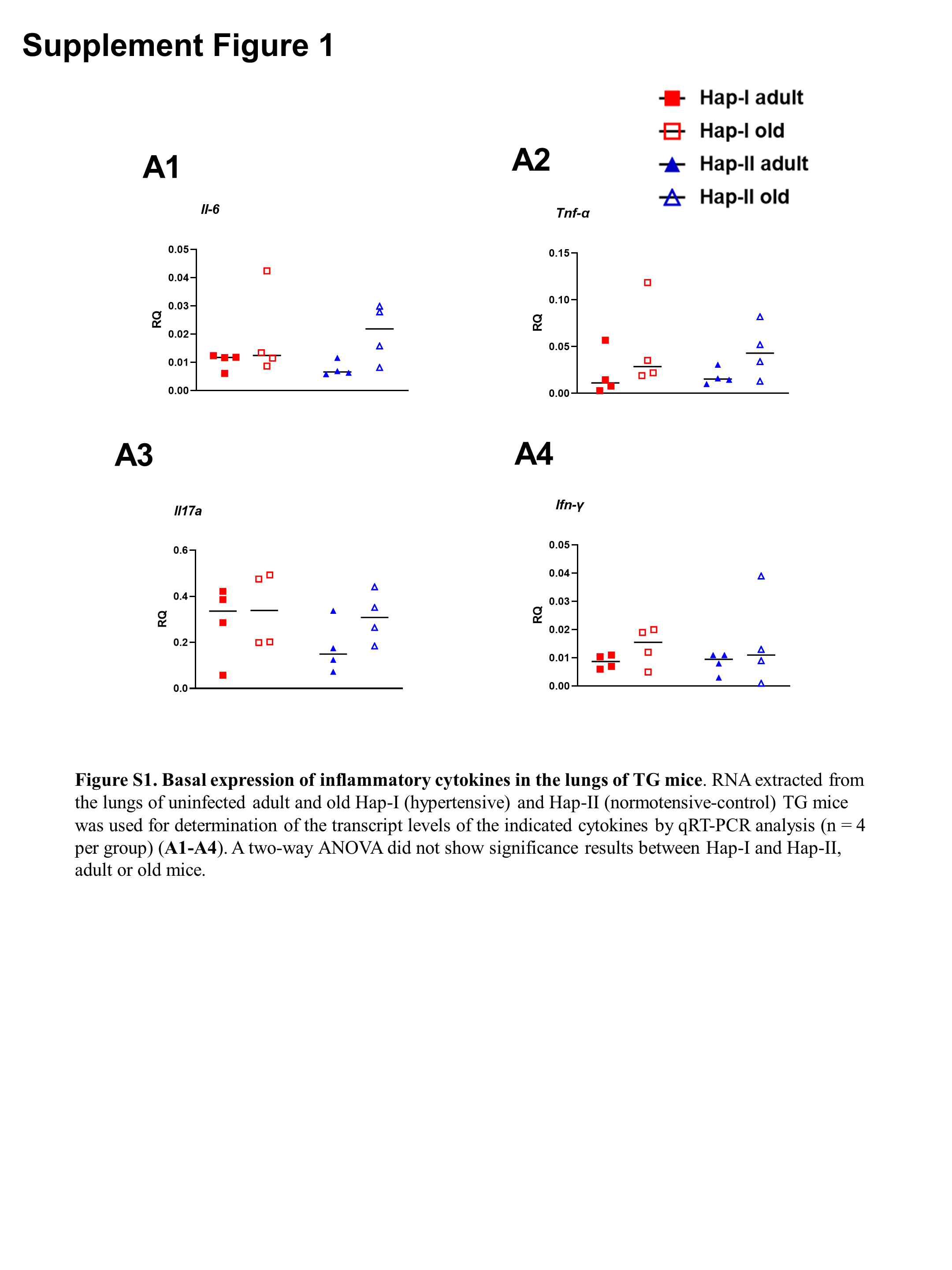

Supplement: Supplementary file 1 [file Image_1.TIF]
